# 

**Published:** 1911-03

**Authors:** 


					CONTENTS.
Page
The Long Fox Lecture: Cerebro-Spinal Syphilis.
By J. Michell
Clarke, M.A., M.D.Cantab., F.R.C.P.
Defects in the Visual Field.
By F. Richardson Cross, M.B.Lond.,
F.R.C.S. ...
33
The Nature and Treatment of Chorea.
By Carey F. Coombs,
M.D., M.R.C.P. Lond..
51
Progress of the Medical Sciences :
Medicine.
R. Shingleton Smith, M.D., B.Sc. Lond.
67
Surgery.
Robert G. Poole Lansdown, M.B., B.S. Dur., t.R.C.S.
73
Ophthalmology.
Cyril H. Walker, M.ts., 13.A. Uantab., f .K.C.S.
77
Reviews of Books
8o
Editorial Notes
88
Notes on Preparations for the Sick
go
The Library of the Bristol Medico-Chirurgical Society   93
Meetings of Societies
95
Obituary Notices
98
Local Medical Not?5v
_ ? \ A I \ \

				

